# Resource management practices of Canadian and American domestic dog breeders and associations with puppy competitive behaviour: A cross-sectional study

**DOI:** 10.1017/awf.2026.10098

**Published:** 2026-07-22

**Authors:** Quinn Rausch, Samantha White, Tina M. Widowski, Jason Coe, Jacquelyn Jacobs, Lee Niel

**Affiliations:** 1 Population Medicine, University of Guelph Ontario Veterinary College, Canada; 2 Veterinärmedizinische Universität Wien Messerli Forschungsinstitut, Austria; 3 Animal Biosciences, University of Guelph, Canada; 4 Animal Science, Michigan State University, United States

**Keywords:** Aggression, animal welfare, *Canis lupus familiaris*, development, resource management, sibling competition

## Abstract

Resource-guarding behaviour is common in companion dogs and negatively impacts human safety, canine welfare, and the human-canine bond. Experiences with resources during critical sensitive periods of development have the potential to influence adult behaviour around resources. Dog breeder management of puppies’ access to resources (e.g. milk, food, and toys) is largely unknown. In this study, dog breeders in Canada and the US (n = 293) were recruited through convenience sampling to complete a cross-sectional survey on breeding and resource management practices and their puppies’ behaviour around resources. Descriptive statistics and logistic regression models were used to determine how resources were managed, the association between management practices and reported competitive behaviour around resources, and the use of aversive-based training methods. Breeder introduction and type of food and toys provided to puppies varied greatly. Most breeders reported intervening during suckling, feeding, and playing to ensure access to resources and habituating puppies to sensory stimuli. Factors associated with increased odds of reported competitive behaviour around food included breeder intervention during suckling and eating, country and breed. Increased odds of reported competitive behaviour around toys was associated with food competition, breeder intervention during play, puppy toy preferences, and whether littermates played together. Increased odds of the use of aversive-based training methods was associated with puppy competitive behaviour during suckling, eating, and playing. This research highlights the need for longitudinal studies on the development of competitive behaviour from birth to sexual maturity and optimal puppy resource management for the prevention of unwanted behaviours in adulthood.

## Introduction

Companion dogs live with 30–50% of Canadians and Americans and are commonly viewed as important members of the family (Dotson & Hyatt 2008; Douzet *et al.* 2023). Understandably, behaviours that are undesirable to humans, including resource guarding, are of concern for most pet caretakers and can negatively impact human health and safety (Reisner & Shofer 2008), animal welfare (Haug 2008) and the human-canine relationship (Hirschman 1994). Resource-guarding behaviour is defined as the use of rapid ingestion, avoidance of interaction, threats or aggression to maintain control of a valuable resource in the presence of a human or non-human animal (Jacobs *et al.* 2018c).

Aggressive resource guarding towards humans was reported in 20% of companion dogs in a study of Eastern Canadian veterinary clientele (Guy *et al.* 2001; Luescher & Reisner 2008). The prevalences of non-aggressive resource-guarding behaviour towards humans and any resource-guarding behaviour towards other non-human animals are likely much higher than 20% but formal prevalence estimates are unavailable. Little is known about the development or causes of resource-guarding aggression, but early experiences are likely to be influential in shaping a dog’s adult relationship with resources. Resource management practices (removing food at meal time), household demographics (multi-dog households), training practices and dog characteristics (higher levels of impulsivity and fear, mixed-breeds, neutered males) have been identified as risk factors for the performance of resource-guarding behaviour, while teaching ‘drop’ and adding palatable food to the bowl were associated with a reduced likelihood of resource-guarding behaviour in a retrospective cross-sectional survey of dog caretakers (Jacobs *et al.* 2018a,b). Jacobs and colleagues (2018a,b) focused on post-adoption/sale experiences of caretakers with adult dogs, but early experiences during critical sensitive periods, can have a large impact on behavioural development, specifically aggression (Scott & Fuller 1965; Haug 2008; Lockwood 2016). Various factors are thought to influence behaviour around resources in the wild (Langenhof & Komdeur 2018), including: the value (Arnott & Elwood 2008) and availability of a resource (Zhu *et al.* 2021), the degree of competition (Range *et al.* 2015; Dale *et al.* 2017; Berghänel *et al.* 2025), an individual’s rank, internal state and subsequent motivation and ability to attain it (Arnott & Elwood 2008; Sarkar *et al.* 2023), and their previous experiences (Hsu & Wolf 1999). A puppy’s early experience accessing different types of valuable resources and performing competitive behaviours (e.g. muzzle and paw pushing) to attain or protect them might impact subsequent resource-guarding behaviour as adults.

Dog breeders are one of the primary sources of companion dogs in Canada and the US and have sole responsibility for puppy development for a large portion (typically 0–8 weeks) of their critical sensitive period (approximately 3–12 weeks). Cutler and colleagues (2017) surveyed Canadian and American puppy adopters/buyers on their socialisation practices after 8 weeks of age and found weak associations between socialisation practices and fearful behaviours at 20 weeks of age. This suggests that a puppy’s earlier experiences during the first eight weeks of life might play a larger role in explaining variation in fearful behaviour in adulthood. Additionally, the use of aversive-based training methods by caretakers to reduce unwanted behaviour in companion dogs is documented to compromise welfare by increasing stress and negative cognitive bias and increases the likelihood of escalation to aggressive behaviour without warning signs (Guilherme Fernandes *et al.* 2017; Ziv 2017; Vieira De Castro *et al.* 2020). Despite the relevance to understanding dog behaviour, little is known about management and training practices used by dog breeders in Canada and the US, specifically regarding resource management. We hypothesise that puppy competitive behaviour in various contexts will be impacted by the way breeders introduce and manage puppies’ access to milk, food and toys, and puppies’ interactions with other littermates and the breeder. Specifically, we predict that putting puppies in competitive situations and intervening in aversive ways during resource interaction will increase the odds of competitive behaviour.

The objectives of this cross-sectional survey of dog breeder resource management practices in the US and Canada are: (1) to determine which resource management practices are common within the Canadian and American dog breeding communities; (2) to determine how closely these practices align with current industry recommendations and scientific literature; (3) to determine factors associated with breeders reporting puppy competitive behaviour towards humans and littermates around food and toys; and (4) to identify factors associated with the use of aversive-based training techniques to deter unwanted behaviour.

## Materials and methods

### Ethical status

All procedures in this study were reviewed and approved by the University of Guelph Research Ethics board for compliance with federal guidelines for research involving human participants (REB# 20-10-002). This research was conducted in 2021 and 2022 during the early, and recovery periods of the COVID-19 pandemic. Breeder resource management practices and breeding and puppy raising more generally might have been impacted during this period, so questions have been added to the survey to address these impacts.

### Participant recruitment

Participation in this questionnaire was restricted to Canadian and American dog caretakers (defined as being responsible for the dog financially) over the age of 18 with at least one female dog that had a litter of puppies within the previous three years (to reduce recall bias while including breeders who bred before the beginning of the COVID-19 pandemic). Snowball sampling techniques were used to promote the survey using social media and email from November 6, 2021, until September 23, 2022. This convenience sampling method relied on referrals of participants to other participants and is utilised to reach groups of people that might not be easily accessible through more traditional means, including the use of random sampling from sampling frames (Biernacki & Waldorf 1981; Atkinson & Flint 2001). Initial online advertisements, including the study description and a link to the survey, were distributed through Facebook, Twitter, Kijiji, Kennel Club listservs (including Canadian, American and United Kennel Clubs), Ontario Veterinary College listservs, Companion Animal Behaviour and Welfare website, International Partnership for Dogs (IPFD) website and listserv and local veterinary clinics. The recruited individuals were also encouraged to share the survey with acquaintances in the breeding community. Incentives were offered for participation with one entry into a draw for four prizes of a $100 CAD (Canadian currency) gift card for survey completion.

### Questionnaire

Hypothesis-driven questions were developed to identify how breeders manage puppies’ access and experience with resources including milk, food, and toys before the puppies are sold. Human, dog, and breeding facility demographics and characteristics were also included in the questionnaire. The development of the questionnaire was informed by current literature on risk factors of resource guarding (Jacobs *et al.* 2018b), fear (Flint *et al.* 2018), aggression (Landsberg *et al.* 2003) and puppy behavioural development (Cornwell & Fuller 1961; Scott & Fuller 1965). The questionnaire was piloted with nine researchers, four breeders and two experts in dog breeding and suggestions were incorporated prior to the launch of the survey. The survey was only available in English and distributed online through Qualtrics (Qualtrics 2020). The final questionnaire consisted of five sections primarily including quantitative multiple-choice questions with some open textboxes: [I] breeder facility demographics (10 questions), [II] feeding practices (20 questions), [III] play management (12 questions), [IV] COVID-19 impacts (2 questions) and [V] breeder demographics (5 questions) (for the full survey, see Supplementary material). Breeders were instructed to answer the questions using their general experience with litters they had raised over the past three years. Participation was anonymous and voluntary, and consent was provided by respondents online prior to accessing the questionnaire.

### Data management

The raw data (n = 363) was reviewed and any responses that did not meet inclusion criteria were removed (n = 70): not from Canada or the US (n = 1), not primary caretaker (n = 4), no female dog that had a litter of puppies within the previous three years (n = 11), answered less than 95% of the survey (n = 8) and were likely to be bot responses (i.e. non-human entries generated by software programmes) (n = 46), based on criteria from (Storozuk *et al.* 2020). Specific breeds were too variable for appropriate inclusion in logistic regression models, so they were combined into the eight breed groups (herding, terrier, toy, sporting, non-sporting, hound, working, other) defined by the Canadian Kennel Club (CKC) for analysis (Canadian Kennel Club 2023). Training methods reported by breeders were categorised into reward-based and aversive-based (Guilherme Fernandes *et al.* 2017). Breeders reported on the performance of competitive behaviour towards humans and littermates around food and toys based on previously defined categories of resource-guarding behaviours in adult dogs including rapid ingestion, avoidance, threats and aggression (Jacobs *et al.* 2018b). Breeders also reported the performance of competitive behaviours observed in puppies during suckling including pushing with paw or muzzle (Rausch *et al.* in review). Targets for competition were separated by species (littermate and human) to be consistent with other literature on resource guarding that distinguishes between dog-dog and dog-human resource guarding (Jacobs *et al.* 2018b).

### Data analysis

Descriptive statistics were generated about the study population, including human demographics, resource management practices (separated by milk, solid food and toy management), dog demographics and dog behaviour and experienced impacts of the COVID-19 pandemic. Continuous variables were described using the mean, median, standard deviation, and range, and categorical variables were described using frequency. For open-ended and textbox responses in ‘other’ categories, content analysis was performed to create codes that represented the content of the answers (Bingham 2023). Five logistic regression models were fitted to estimate the log odds of breeder-reported competitive/resource guarding behaviour: (1) towards humans around food; (2) towards littermates around food; (3) towards humans around toys; and (4) towards littermates around toys; and (5) the use of aversive-based training techniques by breeders. Dichotomous outcomes for each competition model were created with breeders who reported that their puppies displayed any competitive or resource-guarding behaviours in that specific context (i.e. the observation of body blocking, rapid ingestion, avoidance, pushing with nose or paw, threats and/or aggression), were labelled competitive and those who reported no competitive or resource-guarding behaviour in that context were labelled not competitive (except for littermate competition during play which was dichotomised into the presence of threats or aggression and the absence of threats or aggression). All analyses were performed using STATA software (StataCorp 2021) and *P* < 0.05 was considered statistically significant.

To avoid overfitting the models, each outcome was tested only with hypothesis-driven predictors based on available literature and causal diagrams. All models included participant gender, country, average litter size, breed group, and breeding purpose (see Tables 1, 2 and 3 for the categories or form that variables were included in the models). Model [1] estimated the log odds of breeders reporting any ‘*competitive behaviours towards humans around the food bowl’* and the additional tested predictors included suckling intervention, weaning style, solid food presentation, solid food availability, add food into bowl while puppy is eating and remove food bowl while puppy is eating. Model [2] estimated the log odds of breeders reporting any ‘*competitive behaviours towards littermates around the food bowl’* and the additional tested predictors included littermate suckling competition, weaning style, solid food presentation, and solid food availability. Model [3] estimated the log odds of breeders reporting any ‘*competitive behaviours towards humans around toys’* and the additional tested predictors included competition towards humans around the food bowl, toy preference, play together, suckling intervention, toy introduction, toy presentation, toy availability, trade for toys while puppy is playing and remove toy while puppy is playing. Model [4] estimated the log odds of breeders reporting ‘*threatening or aggressive competitive behaviours towards littermates around toys’* and the additional tested predictors included littermate suckling competition, competition towards littermates around the food bowl, toy preference, play together, toy introduction, toy presentation and toy availability. Model [5] estimated the log odds of breeders reporting use of ‘*aversive-based training techniques to deter unwanted behaviours’* and the additional tested predictors included competition towards littermates during suckling, competition towards humans around the food bowl, competition towards humans around toys, education, breeding experience, age, litters per year and litter registration.

**Table 1.** General description of individual and home environment characteristics collected from Canadian and American dog breeder participants (n = 293) in a puppy management survey between 2021 and 2022 Table 1. long description.

Variable	Category	Number (%) or median, mean (± SD), range of respondents
Country	Canada	219/292 (75.0%)
	United States	73/292 (25.0%)
Gender	Woman	264/291 (90.7%)
	Man	26/291 (8.9%)
	Non-binary	1/291 (0.3%)
Age	Years	53.8, 57 (± 15), 21–87
Breeding experience	Years	20.4, 18.5 (± 15), 0–62
Education	High School/College	162/292 (55.5%)
	Bachelors/Graduate School	130/292 (44.5%)
Residence type	Detached house	264/291 (90.7%)
	Townhouse/semi-detached	14/291 (4.8%)
	Apartment/condo	4/291 (1.4%)
	Trailer home	4/291 (1.4%)
	Other	5/291 (1.7%)
Community type	Rural property outside of a concentrated housing area	161/292 (55.1%)
	Village (< 1,000 people)	18/292 (6.2%)
	Small town (1,000–20,000 people)	45/292 (15.4%)
	Large town (20,000–100,000 people)	20/292 (6.8%)
	Small city (100,000–300,000 people)	24/292 (8.2%)
	Large city (300,000–1 million people)	15/292 (5.1%)
	Metropolis (> 1 million people)	9/292 (3.1%)

**Table 2.** General breeding data, dog characteristics and resource management practices reported by Canadian and American dog breeder participants (n = 293) in a puppy management survey between 2021 and 2022 Table 2. long description.

Variable	Category	Number (%) or median, mean (± SD), range of respondents
**Breeding data**		
Breeding intention	Accidental pregnancy of pet	13/292 (4.5%)
	Purposely bred	279/292 (95.5%)
Litters per year	0–2 litters per year	225/277 (81.2%)
	>2 litters per year	52/277 (18.8%)
Litter registration	Yes	218/292 (74.7%)
	No	74/292 (25.3%)
**Dog characteristics**		
Breed Group	Herding	67/292 (22.9%)
	Sporting	67/292 (22.9%)
	Non-sporting	18/292 (6.2%)
	Terrier	29/292 (9.9%)
	Hound	26/292 (8.9%)
	Working	49/292 (16.8%)
	Toy	13/292 (4.5%)
	Other	23/292 (7.9%)
Breeding Purpose	Competitive performance	88/292 (30.1%)
	Not competitive performance	204/292 (69.9%)
Average Litter Size	1–5 Puppies	110/278 (39.6%)
	6–12 Puppies	168/278 (60.4%)
**Food management**		
Weaning style	Left up to the dam	164/292 (56.2%)
	Human intervention	128/292 (43.8%)
Weaning duration	Weeks	2.7, 2 (± 1.5), 0–8
Weaning end	Weeks	7.4, 7 (± 2.1), 2–13
Suckling intervention	Yes	248/293 (84.6%)
	No	45/293 (15.4%)
Solid food introduction	Weeks	4, 4 (± 1.8), 1–9
Type of solid food offered	Wet food only	33/292 (11.3%)
	Wet food mixed with water/milk/formula	38/292 (13.0%)
	Kibble only	2/292 (0.7%)
	Kibble mixed with water/milk/formula	137/292 (46.9%)
	Wet food mixed with kibble	19/292 (6.5%)
	Other (e.g., raw meat, homemade)	63/292 (21.6%)
Solid food availability	1–3 times per day	110/292 (37.7%)
	4 or more times per day or always	182/292 (62.3%)
Solid food presentation	Puppies eat out of individual bowls	43/292 (14.7%)
	Puppies must share bowls	249/292 (85.3%)
Breeder interactions while puppies are eating *(multiple selections possible)*	Add food into bowl	125/293 (42.7%)
	Remove food bowl	72/293 (24.6%)
	Pet or handle puppy	207/293 (70.6%)
	Touch food or feeder	192/293 (65.5%)
	Other	118/293 (40.3%)
Remove food bowl while puppy is eating	Yes	72/292 (24.7%)
	No	220/292 (75.3%)
**Toy management**		
Toy introduction	Weeks	3, 3 (± 1.4), 1–8
Type of toys offered *(multiple selections possible)*	Rope toys	223/293 (76.1%)
	Bone toys for chewing	202/293 (68.9%)
	Balls	240/293 (81.9%)
	Stuffed or plush toys	261/293 (89.1%)
	Toys that squeak or make other noises	269/293 (91.8%)
	Food puzzle or feeder toys	149/293 (50.9%)
	Other (e.g. jungle gym, wobble board, mobile)	78/293 (26.6%)
Toy availability	Toys are always available	259/292 (88.7%)
	Toys available at particular times	33/292 (11.3%)
Toys number	1 or more toys per puppy	226/292 (77.4%)
	Puppies must share toys	66/292 (22.6%)
Breeder interactions while puppies are playing with toys *(multiple selections possible)*	Trade for toy with another toy or food	187/293 (63.8%)
	Remove toy	152/293 (51.9%)
	Pet or handle puppy	261/293 (89.1%)
	Touch toy	225/293 (76.8%)
	Other (e.g. play)	184/293 (62.8%)
**Training methods**		
Aversive (e.g. yell at, hit, slap, spray with water)	Yes	80/292 (27.4%)
	No	212/292 (72.6%)

**Table 3.** Breeder reported puppy behaviour around milk food and toys collected from Canadian and American dog breeder participants (n = 293) in a puppy management survey between 2021 and 2022 Table 3. long description.

Variable	Category	Number (%) of respondents
**Behaviour around food**		
Suckle together	Yes	270/291 (92.8%)
	No	21/291 (7.2%)
Littermate suckling competition	Yes	209/292 (71.6%)
	No	83/292 (28.4%)
Eat together	Yes	282/293 (96.2%)
	No	11/293 (3.8%)
Competition towards humans around the food bowl *(multiple selections possible)*	Body blocking	27/293 (9.2%)
	Rapid ingestion	38/293 (12.9%)
	Avoidance	24/293 (8.2%)
	Pushing with nose or paw	29/293 (9.9%)
	Threats	22/293 (7.5%)
	Aggression	10/293 (3.4%)
	One or more of the above	65/293 (22.2%)
	None of the above	227/293 (77.5%)
Competition towards littermates around the food bowl *(multiple selections possible)*	Body blocking	102/293 (34.8%)
	Rapid ingestion	113/293 (38.6%)
	Avoidance	63/293 (21.5%)
	Pushing with nose or paw	84/293 (28.7%)
	Threats	66/293 (22.5%)
	Aggression	27/293 (9.2%)
	One or more of the above	180/293 (61.4%)
	None of the above	112/293 (38.2%)
**Behaviour around toys**		
Toy Preference	Puppies prefer specific toys	197/292 (67.5%)
	Puppies have no preference for particular toys	95/292 (32.5%)
Play Together	Puppies typically play together	211/287 (73.5%)
	Puppies do not typically play together	76/287 (26.5%)
Competition towards humans around toys *(multiple selections possible)*	Body blocking	32/293 (10.9%)
	Avoidance	97/293 (33.1%)
	Pushing with nose or paw	36/293 (12.3%)
	Threats	41/293 (14.0%)
	Aggression	36/293 (12.3%)
	One or more of the above	124/293 (42.3%)
	None of the above	168/293 (57.3%)
Competition towards littermates around toys *(multiple selections possible)*	Body blocking	158/293 (53.9%)
	Avoidance	223/293 (76.1%)
	Pushing with nose or paw	106/293 (36.2%)
	‘Tug of war’	260/293 (88.7%)
	Threats	144/293 (49.1%)
	Aggression	91/293 (31.0%)
	Threats or aggression	158/293 (53.9%)
	No threats or aggression	132/293 (45.1%)

The general model-building process for all logistic regression models was the same and began with the drawing of a causal diagram including all variables to be tested in the model to determine potential confounding variables and biologically plausible two-way interactions relevant to the relationship between exposures of interest and the outcome. Pairwise correlation among all predictor variables was tested using Pearson Correlation and Spearman’s Rank Correlation (Dohoo *et al.* 2014). If the correlation was > 0.8, the most relevant or accurate variable was kept in the model and the other was excluded (Dohoo *et al.* 2014). Any continuous predictors included in the models were tested for linearity with the log odds of the outcome using locally weighted regression curves (LOWESS) to visually determine the shape of the relationship. If the relationship was non-linear, could not be appropriately modelled with the addition of a quadratic term and could not be transformed, the variable was categorised using biologically relevant categories. Variables meeting a liberal *P*-value of < 0.2 in univariable analysis were considered for inclusion in the multivariate model using backwards stepwise selection. All two-way interactions were tested among or involving remaining main effects in the model and retained if they were statistically significant. Informed through a causal diagram (Dohoo *et al.* 2014), previously tested variables that were potentially causally related to the main effects and the outcome were put back in the model to test if addition caused a greater than 20% change in the coefficients of retained variables, if so, the confounding variable was kept in the models (Dohoo *et al.* 2014). Overall fit of the models was assessed with Pearson and Deviance Chi-square (for binomial data) and Hosmer-Lemeshow (for binary data) Goodness of Fit tests, a non-significant result indicating that the model fit the data. Any observations that were outliers, had abnormally high leverage or were influential on the model or specific cut points were examined by plotting Pearson and Deviance residuals, leverage, Delta-beta, Delta-X^2^ and Delta-Deviance.

## Results

### Participants

A total of 293 survey participants were included in analyses, with them answering questions about themselves, their breeding programme, and their dogs (see Tables 1 and 2 for detailed descriptive statistics). The respondents primarily identified as women (264/291; 90.7%), resided in Canada (219/292; 75.0%), and lived in a detached house (264/291; 90.7%) on a rural property outside of a concentrated housing area (161/291; 55.1%). Most respondents purposely bred (279/292; 95.5%) two or fewer litters per year (225/277; 81.2%) and were registered with a national kennel club (218/292; 74.7%). A variety of dog breeds and breed groups were present with the largest proportion being herding (67/292; 22.9%) and sporting (67/292; 22.9%) dogs.

### Milk management

The majority (248/293; 84.6%) of respondents reported that they intervened with puppies in some way while they were suckling (for detailed descriptive statistics, see Table 2). When invited to expand, the primary reasons for intervention were: [1] ‘*ensuring all puppies had equal access to milk’* whether this was stopping or reducing the need for competitive behaviour, relocating lost or trapped puppies to nipples, or helping smaller puppies by giving them extra time to suckle, stimulating the nipples for them or moving them to breeder-presumed productive nipples. Other reasons for intervening included [2] ‘*exposing puppies to different types of handling’* like petting, stroking, moving or nail trims for habituation and socialisation purposes, [3] ‘*making sure all nipples were in use’* to maintain milk production, [4] ‘*responding to signs of distress’*, for example the aspiration of milk in brachycephalic breeds, and [5] ‘*for weaning purposes’*, interrupting suckling sessions early. Many participants (128/292; 43.8%) reported intervention in the weaning process and the duration of the weaning process ranged from < 1–8 weeks with an average of 2.7 weeks and full separation from the dam ranging from 2–13 weeks of age and averaging at 7.4 weeks of age.

### Solid food management

Survey respondents reported introducing solid food to puppies on average at four weeks of age, but this ranged between one and nine weeks of age (for detailed descriptive statistics, see Table 2). Most respondents (249/292; 85.3%) reported feeding puppies in a manner that required multiple puppies to share one bowl and 62.3% (182/292) had food available four or more times per day, some of which provided free access to food all the time. The type of food fed to puppies varied greatly with 46.9% (137/292) reporting they fed puppies softened kibble mixed with either water, milk, or formula. Interestingly, 21.6% (63/292) of participants selected ‘Other’ and most of these respondents reported feeding their puppies some type of raw meat either on its own or mixed with cow milk, formula, or raw goat milk.

Most respondents also reported that they intervened with puppies in various ways while they were eating, including adding food to the bowl (125/293; 42.7%), petting, or handling the puppy (207/293; 70.6%), touching the food or bowl (192/293; 65.5%) and removing the bowl (72/293; 24.6%) while puppies were still eating. ‘Other’ was also selected by 40.3% (118/293) of participants and the three main themes for other interactions as explained in open textboxes were: [1] ‘*to reorganising puppies to ensure equal access’*, especially to the smaller ones by moving puppies, stopping puppies from stepping in the food bowl or being overly pushy and stopping rapid ingestion, [2] ‘*for habituation purposes’* to different sounds, textures, and interactions like music, clapping, vacuuming or nail trims, and finally [3] ‘*for training purposes’* including specific resource guarding protocols, teaching trade or hand feeding during training sessions.

### Toy management

Respondents reported introducing toys to puppies on average at three weeks of age, ranging from one to eight weeks of age (for detailed descriptive statistics, see Table 2). Most respondents (259/292; 88.7%) always had toys available to the puppies and 77.4% (226/292) provided one or more toys per puppy. A wide variety of toys were provided with the most common being toys that squeak or make noise (269/293; 91.8%) and the least common being food puzzle or feeder toys (149/293; 50.9%). Once again, ‘Other’ was selected by 26.6% (78/293) of respondents and general themes when participants were invited to elaborate included: [1] ‘*larger toy structures’* including play or climbing structures or platforms, [2] ‘*hanging toys’* like mobiles or flirt poles, [3] ‘*children’s toys’* including plastic or wooden blocks, balls, or playground toys, [4] ‘*edible toys’* for example raw bones, or bird wings and [5] ‘*household objects’* meant for habituation to the home environment, including plastic bottles, different surfaces, vacuum, stroller or mirror. Many respondents reported that they intervened with puppies in various ways while they played with toys including trading the puppies for toys using food or another object (187/293; 63.8%), removing toys while the puppies were playing (152/293; 51.9%), petting or handling the puppy (261/293; 89.1%) and touching the toy (225/293; 76.8%). ‘Other’ was selected by 62.8% (184/293) of participants and the main themes from these responses were: [1] ‘*engaging in play with the puppies’* through games like tug of war or fetch, [2] ‘*for training purposes’* including training basic commands, leave it, drop it or trade, and [3] ‘*for habituation purposes’* by desensitising them to different noises, cleaning or talking to the puppies.

### Puppy behaviour around resources

Participants also answered questions about how their puppies typically behaved around milk, food and toys towards littermates and humans (for detailed descriptive statistics, see Table 3). Most respondents (270/291; 92.8%) reported that litters of puppies typically suckled all at the same time and 71.6% (209/292) reported observing competition over nipples between littermates during suckling. Many respondents (180/293; 61.4%) also reported observing competitive behaviour between littermates around the food bowl with rapid ingestion being the most common behaviour observed (113/293; 38.6%) and aggression being the least common (27/293; 9.2%). Competitive behaviour was also reported towards humans around the food bowl, although less common than towards littermates (65/293; 22.2%). Rapid ingestion was also the most common behaviour observed around humans (38/293; 12.9%) and aggression still the least common (10/293; 3.4%). When it came to puppy behaviour around toys, 67.5% (197/292) of respondents reported that individual puppies had preferences for specific toys and 73.5% (211/287) reported that puppies typically played with one another using toys. Competitive behaviour around toys was more common than around food towards both humans (124/293; 42.3%) and littermates (293/293; 100%). The most common behaviour displayed towards littermates was ‘tug of war’ (260/293; 88.7%) and towards humans was avoidance (97/293; 33.1%).

### Food competition towards humans: model [1]

Breeders who reported that they intervened and adjusted puppies during suckling had 0.27 times lower odds of reporting competitive behaviour towards humans around food compared to respondents who did not intervene (*P* < 0.001, 95% CI: 0.13–0.55). Respondents who reported that they regularly added highly palatable food to the food dish while puppies were eating had 1.8 times higher odds of reporting competitive behaviour towards humans around food compared to respondents who did not add food to the food bowl (*P* = 0.047, 95% CI: 1.1–3.3). Lastly, respondents from the US had 2.9 times higher odds of reporting competitive behaviour towards humans around food compared to respondents from Canada (*P* = 0.001, 95% CI: 1.5–5.3). No other tested variables (participant gender, average litter size, breed group, breeding purpose, weaning style, solid food presentation, solid food availability, remove food bowl while puppy is eating) were significantly associated with perceived competitive behaviour towards humans around the food bowl. No observations had abnormally high model influence, leverage or were extreme outliers, Pearson and Deviance Chi-square Goodness of fit test were non-significant indicating the model fit the data (*P* = 0.58), Pseudo R^2^ was low indicating not much variation in the outcome was explained by the data (Pseudo R^2^ = 0.091; for full model details, see Table S1; Supplementary material).

### Food competition towards littermates: model [2]

When investigating factors associated with the performance of any competitive behaviours towards littermates around food, the only significant predictor was breed group categorised into the eight recognised breed groups of the Canadian Kennel Club (2023). When compared to toy breeds, the odds of perceived competitive behaviour towards littermates around food was increased in ‘other’ breeds (primarily mixed breeds) by 8.1 times (*P* = 0.008, 95% CI: 1.7–38), herding breeds by 5.7 times (*P* = 0.008, 95% CI: 1.6–21), working breeds by 4.6 times (*P* = 0.023, 95% CI: 1.2–17) and hound breeds by 4.3 times (*P* = 0.047, 95% CI: 1.1–18). No other tested variables (participant gender, country, average litter size, breeding purpose, littermate suckling competition, weaning style, solid food presentation, and solid food availability) were significantly associated with perceived competitive behaviour between littermates around food. No observations had abnormally high model influence, leverage or were extreme outliers, Pearson and Deviance Chi-square Goodness of fit test were non-significant indicating the model fit the data (*P* = 0.43), Pseudo R^2^ was low indicating not much variation in the outcome was explained by the data (Pseudo R^2^ = 0.045; for full model details, see Table S1; Supplementary material).

### Toy competition towards humans: model [3]

Breeders who reported that they observe competitive behaviour towards humans around the food bowl had 3.4 times higher odds of reporting competitive behaviour towards humans around toys compared to breeders who did not report competition around the food bowl towards humans (*P* < 0.001, 95% CI: 1.9–6.4). Breeders who reported that their puppies typically have preference for specific toys had 2.9 times higher odds of reporting competitive behaviour towards humans around toys compared to puppies with no preference (*P* < 0.001, 95% CI: 1.6–5.2). Breeders who reported that they regularly took toys away from puppies while they were playing had 2.3 times higher odds of reporting competitive behaviour towards humans around toys compared to those who did not regularly take toys away (*P* = 0.002, 95% CI: 1.4–3.8). No other tested variables (participant gender, country, average litter size, breed group, and breeding purpose, play together, suckling intervention, toy introduction, toy presentation, toy availability, trade for toys while puppy is playing) were significantly associated with perceived competitive behaviour towards humans around toys. No observations had abnormally high model influence, leverage or were extreme outliers, Pearson and Deviance Chi-square Goodness of fit test were non-significant indicating the model fit the data (*P* = 0.66), Pseudo R^2^ was low indicating not a lot of variation in the outcome was explained by the data (Pseudo R^2^ = 0.13; for full model details, see Table S1; Supplementary material).

### Toy competition towards littermates: model [4]

Breeders who reported that they observe competitive behaviour between puppies around the food bowl had 3.9 times higher odds of reporting threatening and aggressive competition towards puppies around toys compared to breeders who did not report competition around the food bowl towards puppies (*P* < 0.001, 95% CI: 2.3–6.5). Breeders who reported that puppies typically play together with toys had 0.51 times lower odds of reporting threatening and aggressive competitive behaviour towards puppies around toys compared to puppies who typically play individually with toys (*P* = 0.023, 95% CI: 0.29–0.91). No other tested variables (participant gender, country, average litter size, breed group, breeding purpose, littermate suckling competition, toy preference, toy introduction, toy presentation and toy availability) were significantly associated with perceived competitive behaviour towards littermates around toys. No observations had abnormally high model influence, leverage or were extreme outliers, Pearson and Deviance Chi-square Goodness of fit test were non-significant indicating the model fit the data (*P* = 0.37), Pseudo R^2^ was low indicating not a lot of variation in the outcome was explained by the data (Pseudo R^2^ = 0.091; for full model details, see Table S1; Supplementary material).

### Use of aversive-based training: model [5]

Breeders who reported that they observed competitive behaviour between puppies during suckling had 2.3 times higher odds of reporting the use of aversive-based training techniques to deter unwanted behaviour compared to breeders who did not observe competitive behaviour during suckling (*P* = 0.017, 95% CI: 1.2–4.7). Furthermore, breeders who reported that they observed competitive behaviour towards humans around the food bowl had 3.4 times higher odds (*P* < 0.001, 95% CI: 1.8–6.3), and around toys had 2.3 times higher odds (*P* = 0.004, 95% CI: 1.3–4.0) of reporting the use of aversive-based training techniques to deter unwanted behaviour compared to breeders who did not observe competitive behaviour towards humans around the food bowl and toys, respectively. No other tested variables (participant gender, country, average litter size, breed group, and breeding purpose, education, breeding experience, age, litters per year and litter registration) significantly predicted the use of aversive-based training techniques. No observations had abnormally high model influence, leverage or were extreme outliers, Pearson and Deviance Chi-square Goodness of fit test were non-significant indicating the model fit the data (*P* = 0.60), Pseudo R^2^ was low indicating not a lot of variation in the outcome was explained by the data (Pseudo R^2^ = 0.11; for full model details, see Table S1; Supplementary material).

### COVID-19 pandemic impacts on dog breeding

One-quarter (64/293; 21.8%) of respondents reported that the COVID-19 pandemic had impacted their breeding practices or their access to resources. When asked how puppy introduction and access to resources was impacted, respondents reported a lack of access to specific items including, for example, weaning food, ‘puppy packs’ or chew toys due to [1] ‘*supply chain issues’* because there were none available or stores were closed, [2] ‘*transportation issues’* because items could not be delivered or participants did not have access to a car, [3] ‘*fear’* of going out to stores or other public areas and [4] ‘*financial barriers’* due to rising cost of items in combination with decreased income.

Participants were also asked how COVID-19 impacted their breeding and puppy raising more generally. A small fraction (14/293; 4.7%) of breeders reported starting to breed during the pandemic due to an increased demand for puppies. Of the 21.8% of participants who reported that COVID-19 had impacted them, some discussed impacts on breeding practices more generally and the main themes of these answers included: [1] ‘*not breeding’* during the pandemic, [2] ‘*limited access to desired breeding pairs’* because they could not travel to collect sperm, [3] *‘limited access to socialisation and habituation opportunities for puppies’*, specifically to people and strange environments, due to a lack of visitors, training class and dog show cancellations, and limited ability to leave the property to go on walks or to the pet store, [4] ‘*reduced access to veterinary care’* because veterinary services were further away, closed or less affordable for them and lastly, [5] ‘*transportation difficulties’* meaning that participants could not transport puppies to new homes across borders.

## Discussion

Breeder resource management practices reported in this study are diverse, as expected, and generally align with basic industry and scientific recommendations. The presence of some potentially harmful management practices, such as the use of aversive training techniques and use of early or abrupt weaning, identify an opportunity to prioritise deeper breeder engagement and collaboration regarding current recommendations. Results also confirm that breeder intervention during nursing, eating and play is common, and methods are diverse allowing for the possibility that specific management strategies could modulate the development of behaviour around resources. Results from the five logistic regression models identify a few management strategies (e.g. aversive training techniques, intervention during suckling, group play with toys, adding food to the bowl, removing toys) and dog characteristics (mixed/crossbreeds, preference for toys) that should be investigated more deeply for their relationship with puppy competitive behaviour.

### Milk management

Most breeders reported that they do intervene during suckling, primarily to ensure equal access to milk by assisting smaller littermates. Scramble competition for nipples is common between littermates (Rausch *et al.* in review) and low birth weight individuals have a distinct disadvantage as evidenced by decreased suckling success (Bautista *et al.* 2005) and increased risk of mortality (Mugnier *et al.* 2021) impacting behavioural development (Litten *et al.* 2003; Rödel *et al.* 2008). Breeder codes of practice in Canada and the European Union encourage monitoring and intervening to ensure puppies are getting enough milk (Canadian Veterinary Medical Association 2018; Welfare in Pet Trade 2020), and these interventions are shown to reduce the risk of mortality (Mugnier *et al.* 2021). There are no previous studies evaluating behavioural impacts of breeder intervention, but neonatal puppies are capable of simple associative learning (Cornwell & Fuller 1961) and studies on impacts of gentle handling and high levels of maternal care indicate positive impacts on temperament (Foyer *et al.* 2016; Bray *et al.* 2017) and stress responses (Champagne *et al.* 2008). Breeder intervention during suckling was associated with a decreased odds of perceived competitive behaviour towards humans around the food bowl, perhaps indicating that due to breeder intervention, puppies did not have to perform competitive behaviour as often to access nipples.

Only 43.8% of breeders in the current study reported intervening in some way with the weaning process, which is considerably lower than a previous study from Belgium reporting 63% of breeders intervened in the weaning process by limiting contact with the dam (Dendoncker *et al.* 2019b). This could be explained by the high proportion of very small-scale breeders in this study who might be more likely to wean puppies naturally. Weaning can be a considerable stressor for both the mother and offspring (Santos *et al.* 2020) because it is a frustrating process, associated with changes in food, environment and social dynamics (Grellet *et al.* 2012). The length of the human-managed weaning process varied considerably in our participants with an average of three weeks. Separation of the dam and puppies also varied among participants with most breeders reporting separation between six to eight weeks of age. In feral dogs and wolves, this process takes place gradually between five and ten weeks of age leading to a decrease in the puppy’s maternal bond and an increase in independence and exploratory behaviours (Malm & Jensen 1997; Lord 2013; Pal *et al.* 2021). Large variation in practices between breeders is unsurprising and confirms other reports of inconsistency in breeder management practices (Baublys & Tubelytė 2011; Dendoncker *et al.* 2019b; Blackman *et al.* 2020). Adverse health and behaviour impacts are well documented in pigs and rodents in association with abrupt, pre-mature weaning (Kikusui *et al.* 2006; Campbell *et al.* 2013; Nishi 2020) and these impacts extend to dogs as well (Pierantoni *et al.* 2011). Dog breeding guidelines suggest gradual weaning over three to four weeks and separation only after eight weeks of age (Canadian Veterinary Medical Association 2018; Welfare in Pet Trade 2020).

### Solid food management

The most common type of food offered to puppies was commercial kibble mixed with either water, goat, or cow milk (frequently unpasteurised or ‘raw’), or formula. Commercial diets formulated for weaning and formula specifically for puppies are highly recommended for puppies to ensure adequate nutrition while keeping them satiated (Connolly *et al.* 2014; Canadian Veterinary Medical Association 2018; Welfare in Pet Trade 2020).

Most participants used large communal bowls for the puppies to share during meals. There is minimal knowledge about the impacts of communal vs individual feeding on future behaviour around resources but breeder guidelines recommend feeding in separate bowls to deter competition and ensure all puppies are eating the correct amount of food (Canadian Veterinary Medical Association 2018; Welfare in Pet Trade 2020). Studies on cofeeding in free-ranging dogs show that group foraging, compared to solitary feeding, is associated with more rapid ingestion, particularly when patches are high in value but too large to be effectively monopolised by a single individual (Sarkar *et al.* 2023; Berghänel *et al.* 2025). Small food patches are almost always monopolised by one dog, resulting in little or no cofeeding but at larger food patches, aggression initially increases as additional competitors join but then declines as feeding group size grows (Sarkar *et al.* 2023; Berghänel *et al.* 2025). This indicates that the energetic and social costs of aggression outweigh the benefits of attempting monopolisation once control becomes difficult leading dogs to shift from contest competition (e.g. aggression) to scramble competition (e.g. rapid ingestion or avoidance) as the optimal foraging strategy (Berghänel *et al.* 2025). With the predominant feeding style for breeders being a few communal bowls filled with high calorie, high value puppy food, rapid ingestion might be inadvertently rewarded, especially if the group size is high enough to deter monopolisation. Breeders reported whether they fed puppies individually or communally but due to variation in litter size and puppy size, feeder space per puppy could not be determined. It is possible that feeder space is associated with competitive behaviour more so than the number of feeders.

Although 58% of breeders reported that their puppies displayed no behaviour around the food bowl that required intervention, only 38.2% of breeders reported no competitive behaviour in the same context. This indicates that a certain level of competition was deemed acceptable by some participants. Depending on an individual puppy’s experience with this competition during eating, this could have varying impacts on behavioural development. Studies evaluating the impacts of early life stress indicate that intense or prolonged stress during the neonatal (birth to 2 weeks of age) or transitional period (2 to 4 weeks of age), for example, prolonged maternal separation, might increase stress sensitivity in adults leading to increased use of aggression (Veenema 2009; Nishi 2020). This suggests that very stressful competitive situations around food at a young age might predispose puppies to aggressive resource guarding later in life. On the other hand, mild or moderate stressors, including brief maternal separation or daily gentle handling, seem to have a protective effect on stress resilience. Specifically in puppies, exposure to varied stimuli and handling between birth and 5 weeks of age had positive impacts on confidence, exploration and social dominance (Gazzano *et al.* 2008). This suggests that if competitive stress around the food bowl is mild, it might habituate puppies to low levels of competition, especially if access to an appropriate amount of food is not hindered and increase their resilience to stress during future competitive situations.

Breeders in this study reported interacting with puppies in various ways during eating, including but not limited to adding food into the bowl and taking the food bowl away. In older dogs, caretakers reporting that they regularly add food to the food bowl has been associated with a decreased risk of resource-guarding behaviour while regular removal the food bowl is associated with an increased risk of resource-guarding behaviour (Jacobs *et al.* 2018b). General recommendations caution against the removal of the food bowl and handling of the dogs during eating and instead recommend habituating dogs positively to human approach around food by adding food into the bowl during eating (Landsberg *et al.* 2003; Overall 2013). When it comes to puppies, early habituation to handling and touch as well as socialisation is very important and should be a positive experience for the puppy (Howell *et al.* 2015; Vaterlaws-Whiteside & Hartmann 2017; Welfare in Pet Trade 2020). If handling of the puppies during eating causes distress or early signs of resource guarding, like rapid ingestion, further handling could potentially exacerbate the behaviour. In contrast to results from Jacobs and colleagues (2018b), breeders in the current study who reported adding food to the food bowl during a meal had an increased odds of reporting that their puppies displayed competitive behaviour towards humans at the food bowl. Due to the cross-sectional nature of the study, we are unable to infer causation, but it is possible this association is caused by the breeder noticing competition in their puppies and proactively attempting to decrease its prevalence by pairing human approach with a tasty treat (a resource guarding treatment protocol). Alternatively, adding a highly palatable food to the food bowl might also increase a puppy’s motivation or interest to consume the food quickly, which could be interpreted as rapid ingestion even though the motivation is not to resource guard. In the current study, although brief descriptions of behaviours were provided in survey questions, no testing was carried out to determine participants’ accuracy in identifying described behaviours. Interpretation of non-aggressive resource-guarding behaviour is highly contextual and requires objective measures of behaviour like video recordings or live observations (Jacobs *et al.* 2017).

### Toy management

Many participants reported providing a wide variety of toys. One-quarter of participants even listed numerous additional objects in the ‘Other’ category primarily including different household or sensory objects for habituation. There was a range of reported time for introduction of toys indicating that some breeders at the upper end of this range might have missed valuable time to get puppies habituated to different sensory experiences, interactions and play behaviours before they go home to new caretakers. Literature on critical periods of early development emphasises the importance of positively exposing and habituating puppies to a variety of different sensory experiences between 3 and 12 weeks of age to reduce the risk of fear and aggression towards novelty (Scott & Fuller 1965; Howell *et al.* 2015; Vaterlaws-Whiteside & Hartmann 2017). Breeder guidelines similarly recommend providing an enrichment programme that includes exercise areas with toys, paddling pools, agility equipment and raised platforms to stimulate different types of activity including play (Canadian Veterinary Medical Association 2018; Welfare in Pet Trade 2020). Most respondents reported that toys were always available and that the number of toys offered allowed puppies to all have at least one toy to themselves if desired. This abundance might be protective against competitive behaviour around toys by avoiding resource scarcity as seen in studies on food abundance and competition (Arnott & Elwood 2008; Zhu *et al.* 2021). The value an individual assigns to a toy may depend on the puppy’s own preference. In this study, 67% of respondents reported that puppies favoured specific toys, and these preferences were linked to increased odds of perceived competitive behaviour toward humans around toys. This aligns with previous literature showing that the value an individual places on a resource influences competitive behaviour to obtain and retain it (Arnott & Elwood 2008; Benhaiem *et al.* 2013). Alternatively, breeders who notice that puppies have preferences for specific toys could be more observant of puppy behaviour in general and therefore more likely to notice and report competitive behaviour as well.

Most participants reported interacting in a variety of ways with puppies during play. Breeding guidelines encourage breeders to positively interact with dogs during exercise through play and reward-based training and avoid forcing the dog to interact if they do not wish to (Canadian Veterinary Medical Association 2018; Welfare in Pet Trade 2020). Some participants reported frequently removing the toy from the puppy during play and this practice was associated with an increased odds of perceived competitive behaviour towards humans around toys. Removing toys could create a negative association with human interaction during play through associative learning and might be a risk factor for further resource guarding behaviour (Jacobs *et al.* 2018b). The removal of toys and perceived puppy competitive behaviour in response could also just be ‘part of the game’ and not of concern for breeders. Other breeders reported teaching ‘drop’ using reward-based training or offering a trade of food or another high value object for a toy that a puppy was playing with, which has been associated with decreased odds of resource guarding towards humans and other dogs (Jacobs *et al.* 2018b) and is frequently used as a preventive and early treatment method for resource-guarding behaviour (Landsberg *et al.* 2003; Overall 2013). Interestingly, we found no association between breeders who reported teaching ‘drop’ or ‘trade’ commands and competitive behaviour. It is possible that although breeders are attempting to teach these tasks, the puppies are too young to fully master them and therefore impacts on competitive behaviour are not present. Alternatively, breeders might not exclusively be training puppies using reward-based methods. This is exemplified by the positive association that was seen between aversive-based training methods to deter unwanted behaviour and competitive behaviour towards humans during suckling, around food and around toys. Aversive training methods could encourage competitive behaviour towards humans but these associations could also be explained by unwanted behaviour leading to increased likelihood of caretakers employing aversive training methods (Hirschman 1994). Perhaps breeders who were seeing competitive behaviour were more likely to then employ more aversive training methods as a result.

Puppies also frequently interact with littermates around toys and breeder management of these interactions might play a role in these experiences. Just under half of breeders reported that puppies did not display behaviours during play that they needed to discourage; however, almost all breeders reported some level of competitive behaviour with over half reporting threats and aggression towards littermates indicating once again that some level of competitive behaviour is deemed acceptable between littermates. Like other types of socialisation, interactions around toys should generally be positive for the puppy to decrease the likelihood of fear in the moment and in similar situations later in life (Vaterlaws-Whiteside & Hartmann 2017; Canadian Veterinary Medical Association 2018; Welfare in Pet Trade 2020). A puppy’s individual experience and subsequent behaviour might vary depending on their threshold for fear, motivation for and value of toys and temperament differences like impulsivity and boldness (Jacobs *et al.* 2018a,b).

Breeders who reported that their puppies typically played all together showed reduced odds of reporting competitive behaviour towards littermates. This is potentially because social play tends to end if aggressive behaviour goes too far so the cessation of play following aggressive competition might reduce the likelihood of this behaviour, a form of associative learning called negative punishment (Guilherme Fernandes *et al.* 2017). This might also indicate that puppies who play individually value specific toys more highly or might not be interested in social play and are therefore more likely to display competitive behaviours to deter interaction.

### Consistency of competitive behaviour across context

Consistency of competitive behaviour reported across different resource contexts is observed in the present study through the results of logistic regression models where the presence of competitive behaviour towards humans around food significantly predicted competitive behaviour towards humans around toys. Similarly, perceived competitive behaviour towards littermates around food significantly predicted competitive behaviour towards littermates around toys. Consistency across context might indicate some puppies have a predisposition for competitiveness or that some puppies have learned that competitive behaviour is successful and therefore generalise its employment in other situations (Huntingford & Turner 1987). Moderate consistency in competitive tendencies have been demonstrated by Wright (1980) through bone competition tests where the percentage of time each puppy spent monopolising or sharing the bone were relatively consistent. Alternatively, as these are breeder level data, this finding could reflect that breeders who report observing competition in one situation are simply more observant, more concerned about competitive behaviour or more versed on puppy body language and are therefore more likely to report seeing it in other contexts as well.

### Influences of breed group

Breed group was a significant predictor for perceived competitive behaviour towards littermates around food. Specifically, ‘other’ breeds, which were made up of mixed breeds and crossbreeds, had the highest odds of displaying competitive behaviour around the food bowl to other littermates out of all breed groups with four of the eight breed groups (herding, hound, working, ‘other’) having significantly increased odds when compared with toy breeds. Considerable literature supports the role of genetics in behaviour predisposition whether this is artificial selection for aggression, prey drive, food drive in particular breeds due to their intended purpose or genetic variation in all traits in general (Passalacqua *et al.* 2011; Udell *et al.* 2014). Mixed breeds having higher odds of perceived competitive behaviour aligns with the findings of Jacobs and colleagues (2018a,b), who reported mixed-breed dogs as a risk factor for resource guarding both towards humans and people. Mixed-breed dogs in the general population are more likely to be acquired at an older age from shelters than bred purposefully, suggesting a higher chance of aversive early-life experiences that could explain increased prevalence of resource guarding. In this study, mixed-breed litters might have been accidental or produced by less-experienced breeders with fewer standardised practices. In contrast, breeders of pure breeds might be more likely to follow management practices associated with the dog’s intended purpose, Kennel Club breed standards, or peer recommendations, which could confound the genetic influences on behaviour. It should be noted that the confidence intervals for these effect sizes were large, indicating uncertainty in the model likely caused by sparse data for some breed groups and this result should be interpreted with caution.

### Influences of country

The higher reporting of food-related competitive behaviour towards humans by breeders in the US might be due to differing management practices associated with competition or reflect differences in interpretation of the survey questions, ability to identify puppy behaviour or social desirability. If American breeders have a lower threshold for reporting behaviours as competitive or are more familiar with identifying and discussing early behavioural concerns, they might be more likely to report the presence of competitive behaviour. Alternatively, this finding could reflect a difference in dog management practices that influence puppy behaviour. American dog breeders must be licensed through the United States Department of Agriculture (Animal and Plant Health Inspection Service 2023) but Canada does not have a federal breeder licence requirement and many of these regulations are left up to the province or municipality. It is possible that requirements under the licence also influence puppy resource management in a way that increases the presence or recognition of competition. Finally, this finding could be the result of differences in social desirability bias that are seen across cultures (Bernardi & Guptill 2008). Canadians might have smaller, more interconnected breeder communities or increased concern about their reputation and therefore be more likely to underreport undesirable behaviours.

### Study limitations

This study has several limitations that were mitigated where possible through methodological means but should be taken into consideration when interpreting the results. Selection bias might be present due to the nature of a cross-sectional survey and the use of convenience sampling. Participants might be statistically different (e.g. more interested in behaviour, more committed to breeding or more likely to answer in a certain way) than non-participants. Additionally, due to the timing of this survey, data collection occurred during the COVID-19 pandemic, which potentially influenced breeding practices and limited data collection without in-person observation. To account for this, information on COVID-19’s impacts on breeding practices and resource availability was collected and the overall survey responses were used to generate hypotheses to guide future longitudinal studies, not to infer causality.

Our sample consists predominantly of women who are small-scale breeders in Canada living on a rural property, which limits the generalisability of our results to the broader population of dog breeders in Canada and the US. Limits to generalisability might be particularly true for large-scale dog breeders that are documented to have different management practices than small-scale, in-home breeders (Dendoncker *et al.* 2019a). The findings of the present study are discussed in the context of the population that participated and are intended to generate hypotheses for origins of competitive behaviour. This is intended to inform future larger scale studies with more diverse populations that are more representative of the whole dog breeding community or more homogeneous populations representative of certain management practices.

Recall and social desirability biases might also be present because participants were asked to self-report via online survey. Participants have the possibility of being incorrect in their recollection of their management practices or puppy behaviour and therefore incorrectly answer questions. Participants might also not want to answer certain questions truthfully due to fear of judgement or a desire to answer the questions in a way that helps the researchers with the objectives of the study. These biases were minimised by limiting participation in the study to breeders that had had a litter within the past three years and instructing them to discuss general experiences over the last three years and by not sharing the specific objectives and hypotheses of the study with participants.

Finally, this study measured breeder level data about participant experience with puppy behaviour over the past three years and these data cannot be used to make claims about individual puppies or even litters. Furthermore, variables were often dichotomised for inclusion into logistic regression models resulting in a lack of nuance regarding occasional/chronic or mild/severe behaviours. This likely resulted in an overestimation of prevalence due to increased sensitivity and an over- or under-estimation of true associations. This limitation is mitigated through interpretation of data purely at the breeder level and the associations purely for hypothesis generation. Further research should investigate these associations more specifically at the puppy and litter level using objective measures of behaviour rather than breeder reports, which we were limited to in this study due to COVID-19 restrictions, and whether there are other factors that might be residually confounding the data, as indicated by the low predictive ability of all logistic regression models.

### Animal welfare implications

This study highlights how early resource management practices used by dog breeders may shape puppies’ experiences with valuable resources. We identified several management practices that do not align with scientific and industry standards and pose a welfare risk such as the use of aversive training techniques, abrupt or early weaning, and potentially, encouragement of competitive behaviour. Variability in timing, type, and handling of resources, combined with intervention strategies that either encourage or discourage competition, contribute to a puppy’s affective experience while at the breeding facility. These early experiences also have the potential to influence development of adult behavioural outcomes, such as resource guarding that might put dogs at risk of inhumane training methods, relinquishment, and euthanasia. This research generates hypotheses surrounding resource management that sets the groundwork for longitudinal research exploring optimal resource management for puppies.

## Conclusion

This is the first study to examine dog breeder resource management practices and associations with puppy competitive behaviour around resources. We found that management practices varied greatly across participants, including the timelines for introduction, the availability of resources, and the type of resources provided. Breeders predominantly reported intervening during interaction with resources both in ways that may encourage and discourage competition. This study also highlights various management factors that were found to be associated with increased odds of reported competitive behaviour. These results generate hypotheses for management practices that may encourage or discourage competitive behaviour in different resource contexts but causal links between management practices, early competitive behaviours and resource guarding behaviour still need to be established. Future longitudinal studies following individual puppies from birth to adulthood to determine optimal levels of competition and management practices for resource guarding prevention are necessary.

## Supporting information

10.1017/awf.2026.10098.sm001 Rausch et al. supplementary material Rausch et al. supplementary material

## Supplementary material

The supplementary material for this article can be found at http://doi.org/10.1017/awf.2026.10098.

## Long descriptions

### Table 1. Long description

The table consists of three columns: Variable, Category, and Number (percentage) or median, mean plus or minus S D, range of respondents.

* Country: Canada accounts for 219/292 (75.0 percent) and United States for 73/292 (25.0 percent).

* Gender: Woman 264/291 (90.7 percent), Man 26/291 (8.9 percent), and Non-binary 1/291 (0.3 percent).

* Age: Median 53.8, mean 57 plus or minus 15, range 21 to 87 years.

* Breeding experience: Median 20.4, mean 18.5 plus or minus 15, range 0 to 62 years.

* Education: High School or College 162/292 (55.5 percent), Bachelors or Graduate School 130/292 (44.5 percent).

* Residence type: Detached house 264/291 (90.7 percent), Townhouse or semi-detached 14/291 (4.8 percent), Apartment or condo 4/291 (1.4 percent), Trailer home 4/291 (1.4 percent), and Other 5/291 (1.7 percent).

* Community type: Rural property 161/292 (55.1 percent), Village less than 1,000 people 18/292 (6.2 percent), Small town 1,000 to 20,000 people 45/292 (15.4 percent), Large town 20,000 to 100,000 people 20/292 (6.8 percent), Small city 100,000 to 300,000 people 24/292 (8.2 percent), Large city 300,000 to 1 million people 15/292 (5.1 percent), and Metropolis greater than 1 million people 9/292 (3.1 percent).

Navigate back to Table 1..

### Table 2. Long description

The table is divided into four primary sections:

1. Breeding data:

- Breeding intention: 95.5% purposely bred, 4.5% accidental.

- Litters per year: 81.2% have 0 to 2 litters; 18.8% have more than 2.

- Litter registration: 74.7% yes, 25.3% no.

2. Dog characteristics:

- Breed Group: Herding and Sporting are highest at 22.9% each, followed by Working at 16.8%, Terrier at 9.9%, Hound at 8.9%, Non-sporting at 6.2%, and Toy at 4.5%.

- Breeding Purpose: 69.9% not competitive performance, 30.1% competitive performance.

- Average Litter Size: 60.4% have 6 to 12 puppies; 39.6% have 1 to 5 puppies.

3. Food management:

- Weaning style: 56.2% left up to the dam, 43.8% human intervention.

- Weaning duration: Median 2.7 weeks, mean 2 plus or minus 1.5, range 0 to 8.

- Weaning end: Median 7.4 weeks, mean 7 plus or minus 2.1, range 2 to 13.

- Solid food introduction: Median 4 weeks, mean 4 plus or minus 1.8, range 1 to 9.

- Type of solid food: Kibble mixed with water/milk/formula is most common at 46.9%, followed by other (raw/homemade) at 21.6%.

- Presentation: 85.3% of puppies must share bowls.

- Breeder interactions: 70.6% pet or handle puppy, 65.5% touch food or feeder, 42.7% add food, 24.6% remove food bowl.

4. Toy management:

- Toy introduction: Median 3 weeks, mean 3 plus or minus 1.4, range 1 to 8.

- Type of toys: Squeaky toys (91.8%) and stuffed/plush toys (89.1%) are most common, followed by balls (81.9%), rope toys (76.1%), and bone toys (68.9%).

Navigate back to Table 2..

### Table 3. Long description

The table is organized into three columns: Variable, Category, and Number (percentage) of respondents.

Section 1: Behaviour around food.

- Suckle together: Yes 92.8 percent; No 7.2 percent.

- Littermate suckling competition: Yes 71.6 percent; No 28.4 percent.

- Eat together: Yes 96.2 percent; No 3.8 percent.

- Competition towards humans around the food bowl: 77.5 percent reported none. Competitive behaviors included rapid ingestion at 12.9 percent, pushing at 9.9 percent, body blocking at 9.2 percent, avoidance at 8.2 percent, threats at 7.5 percent, and aggression at 3.4 percent.

- Competition towards littermates around the food bowl: 61.4 percent reported one or more behaviors. Top behaviors were rapid ingestion at 38.6 percent, body blocking at 34.8 percent, and pushing at 28.7 percent.

Section 2: Behaviour around toys.

- Toy Preference: 67.5 percent prefer specific toys; 32.5 percent have no preference.

- Play Together: 73.5 percent typically play together; 26.5 percent do not.

- Competition towards humans around toys: 57.3 percent reported none. Avoidance was the most common behavior at 33.1 percent, followed by threats at 14.0 percent.

- Competition towards littermates around toys: Tug of war was the most frequent behavior at 88.7 percent, followed by avoidance at 76.1 percent, body blocking at 53.9 percent, and threats or aggression at 53.9 percent.

Navigate back to Table 3..

## References

[r1] Animal and Plant Health Inspection Service 2023 Licensing and Registration Under the Animal Welfare Act: Guidelines for Dealers, Exhibitors, Transporters, and Researchers. United States Department of Agriculture. https://www.aphis.usda.gov/sites/default/files/graybook.pdf (accessed 29th May 2026).

[r2] Arnott G and Elwood R 2008 Information gathering and decision making about resource value in animal contests. Animal Behaviour 76: 529–542. 10.1016/j.anbehav.2008.04.019

[r3] Atkinson R and Flint J 2001 Accessing Hidden and Hard-to-Reach Populations: Snowball Research Strategies. Social Research Update 33.

[r4] Baublys V and Tubelytė V 2011 Determining breeding knowledge, goals and practices of Lithuanian dog breeders. Biologija 57(1). 10.6001/biologija.v57i1.870

[r5] Bautista A, Mendoza-Degante M, Coureaud G, Martínez-Gómez M and Hudson R 2005 Scramble competition in newborn domestic rabbits for an unusually restricted milk supply. Animal Behaviour 70(5): 1011–1021. 10.1016/j.anbehav.2005.01.015

[r6] Benhaiem S, Hofer H, Dehnhard M, Helms J and East ML 2013 Sibling competition and hunger increase allostatic load in spotted hyaenas. Biology Letters 9(3): 20130040. 10.1098/rsbl.2013.0040 23616643 PMC3645033

[r7] Berghänel A, Lazzaroni M, Ferenc M, Pilot M, El Berbri I, Marshall-Pescini S and Range F 2025 Cofeeding at rich clumped food patches in free-ranging dogs: social tolerance or scramble competition? Behavioral Ecology and Sociobiology 79(4): 51. 10.1007/s00265-025-03590-8 40242211 PMC11996968

[r8] Bernardi RA and Guptill ST 2008 Social Desirability Response Bias, Gender, and Factors Influencing Organizational Commitment: An International Study. Journal of Business Ethics 81(4): 797–809. 10.1007/s10551-007-9548-4

[r9] Biernacki P and Waldorf D 1981 Snowball Sampling: Problems and Techniques of Chain Referral Sampling. Sociological Methods & Research 10(2): 141–163. 10.1177/004912418101000205

[r10] Bingham AJ 2023 From Data Management to Actionable Findings: A Five-Phase Process of Qualitative Data Analysis. International Journal of Qualitative Methods 22: 1–11. 10.1177/16094069231183620

[r11] Blackman SA, Wilson BJ, Reed AR and McGreevy PD 2020 Reported Motivations and Aims of Australian Dog Breeders—A Pilot Study. Animals 10(12): 2319. 10.3390/ani10122319 33297412 PMC7762288

[r12] Bray EE, Sammel MD, Cheney DL, Serpell JA and Seyfarth RM 2017 Characterizing Early Maternal Style in a Population of Guide Dogs. Frontiers in Psychology 8. 10.3389/fpsyg.2017.00175 PMC5301023 28239365

[r13] Campbell JM, Crenshaw JD and Polo J 2013 The biological stress of early weaned piglets. Journal of Animal Science and Biotechnology 4(1): 19. 10.1186/2049-1891-4-19 23631414 PMC3651348

[r14] Canadian Kennel Club 2023 *CKC Breed Standards.* https://www.ckc.ca/en/Events/CKC-Breed-Standards (accessed 29th May 2026).

[r15] Canadian Veterinary Medical Association 2018 *A code of practice for Canadian kennel operations.* Canadian Veterinary Medical Association. https://www.canadianveterinarians.net/media/jgkpjouk/a-code-of-practice-for-canadian-kennel-operations-3rd-edition-2018.pdf (accessed 29th May 2026).

[r16] Champagne DL, Bagot RC, Van Hasselt F, Ramakers G, Meaney MJ, De Kloet ER, Joels M and Krugers H 2008 Maternal Care and Hippocampal Plasticity: Evidence for Experience-Dependent Structural Plasticity, Altered Synaptic Functioning, and Differential Responsiveness to Glucocorticoids and Stress. Journal of Neuroscience 28(23): 6037–6045. 10.1523/JNEUROSCI.0526-08.2008 18524909 PMC6670331

[r17] Connolly KM, Heinze CR and Freeman LM 2014 Feeding practices of dog breeders in the United States and Canada. Journal of the American Veterinary Medical Association 245(6): 669–676. 10.2460/javma.245.6.669 25181271

[r18] Cornwell AC and Fuller JL 1961 Conditioned responses in young puppies. Journal of Comparative and Physiological Psychology 54(1): 13–15. 10.1037/h0043659 24545541

[r19] Cutler JH, Coe JB and Niel L 2017 Puppy socialization practices of a sample of dog owners from across Canada and the United States. Journal of the American Veterinary Medical Association 251(12): 1415–1423. 10.2460/javma.251.12.1415 29190195

[r20] Dale R, Range F, Stott L, Kotrschal K and Marshall-Pescini S 2017 The influence of social relationship on food tolerance in wolves and dogs. Behavioral Ecology and Sociobiology 71(7): 107. 10.1007/s00265-017-2339-8 28725102 PMC5493712

[r21] Dendoncker P, De Keuster T, Diederich C, Dewulf J and Moons CPH 2019a On the origin of puppies: breeding and selling procedures relevant for canine behavioural development. Veterinary Record 184(23): 710–710. 10.1136/vr.104979 30696712

[r22] Dendoncker P-A, De Keuster T, Diederich C, Dewulf J and Moons CPH 2019b On the origin of puppies: breeding and selling procedures relevant for canine behavioural development. Veterinary Record 184(23): 710–710. 10.1136/vr.104979 30696712

[r23] Dohoo I Robert, Martin SW and Stryhn H 2014 Veterinary Epidemiologic Research, Second *Edition.* Ver Inc: Charlotte, Canada.

[r24] Dotson MJ and Hyatt EM 2008 Understanding dog–human companionship. Journal of Business Research 61: 457–466. 10.1016/j.jbusres.2007.07.019

[r25] Douzet A, Brooks D, Santos E, Cairns L, Brandao L, Enders M-J, Chuckekamrai S, Ryan S and Dohne W 2023 Global State of Pet Care Stats, Facts and Trends. Health for Animals. https://www.healthforanimals.org/wp-content/uploads/2022/07/Global-State-of-Pet-Care.pdf (accessed 29th May 2026).

[r26] Flint HE, Coe JB, Serpell JA, Pearl DL and Niel L 2018 Identification of fear behaviors shown by puppies in response to nonsocial stimuli. Journal of Veterinary Behavior 28: 17–24. 10.1016/j.jveb.2018.07.012

[r27] Foyer P, Wilsson E and Jensen P 2016 Levels of maternal care in dogs affect adult offspring temperament. Scientific Reports 6(1): 19253. 10.1038/srep19253 26758076 PMC4725833

[r28] Gazzano A, Mariti C, Notari L, Sighieri C and McBride EA 2008 Effects of early gentling and early environment on emotional development of puppies. Applied Animal Behaviour Science 110(3–4): 294–304. 10.1016/j.applanim.2007.05.007

[r29] Grellet A, Feugier A, Chastant-Maillard S, Carrez B, Boucraut-Baralon C, Casseleux G and Grandjean D 2012 Validation of a fecal scoring scale in puppies during the weaning period. Preventive Veterinary Medicine 106(3–4): 315–323. 10.1016/j.prevetmed.2012.03.012 22520179 PMC7114323

[r30] Guilherme Fernandes J, Olsson IAS and Vieira De Castro AC 2017 Do aversive-based training methods actually compromise dog welfare?: A literature review. Applied Animal Behaviour Science 196: 1–12. 10.1016/j.applanim.2017.07.001

[r31] Guy NC, Luescher UA, Dohoo SE, Spangler E, Miller JB, Dohoo IR and Bate LA 2001 A case series of biting dogs: characteristics of the dogs, their behaviour, and their victims. Applied Animal Behaviour Science 74(1): 43–57. 10.1016/S0168-1591(01)00155-1

[r32] Haug LI 2008 Canine Aggression Toward Unfamiliar People and Dogs. Veterinary Clinics of North America: Small Animal Practice 38(5): 1023–1041. 10.1016/j.cvsm.2008.04.005 18672152

[r33] Hirschman EC 1994 Consumers and Their Animal Companions. Journal of Consumer Research 20(4): 616. 10.1086/209374

[r34] Howell T, King T and Bennett P 2015 Puppy parties and beyond: the role of early age socialization practices on adult dog behavior. Veterinary Medicine: Research and Reports 143. 10.2147/VMRR.S62081 PMC6067676 30101101

[r35] Hsu Y and Wolf LL 1999 The winner and loser effect: integrating multiple experiences. Animal Behaviour 57(4): 903–910. 10.1006/anbe.1998.1049 10202098

[r36] Huntingford F and Turner A 1987 Patterns in Animal Conflict. Chapman and Hall: London, UK.

[r37] Jacobs JA, Coe JB, Pearl DL, Widowski TM and Niel L 2018a Factors associated with canine resource guarding behaviour in the presence of dogs: A cross-sectional survey of dog owners. Preventive Veterinary Medicine 161: 134–142. 10.1016/j.prevetmed.2017.02.004 28274585

[r38] Jacobs JA, Coe JB, Pearl DL, Widowski TM and Niel L 2018b Factors associated with canine resource guarding behaviour in the presence of people: A cross-sectional survey of dog owners. Preventive Veterinary Medicine 161: 143–153. 10.1016/j.prevetmed.2017.02.005 28268035

[r39] Jacobs JA, Coe JB, Widowski TM, Pearl DL and Niel L 2018c Defining and Clarifying the Terms Canine Possessive Aggression and Resource Guarding: A Study of Expert Opinion. Frontiers in Veterinary Science 5: 115. 10.3389/fvets.2018.00115 29942810 PMC6004413

[r40] Jacobs JA, Pearl DL, Coe JB, Widowski TM and Niel L 2017 Ability of owners to identify resource guarding behaviour in the domestic dog. Applied Animal Behaviour Science 188: 77–83. 10.1016/j.applanim.2016.12.012

[r41] Kikusui T, Nakamura K, Kakuma Y and Mori Y 2006 Early weaning augments neuroendocrine stress responses in mice. Behavioural Brain Research 175(1): 96–103. 10.1016/j.bbr.2006.08.007 16959332

[r42] Landsberg G, Hunthausen W and Ackerman L 2003 Handbook of Behaviour Problems of the Dog and Cat, Second *Edition.* Saunders: New York, NY, USA.

[r43] Langenhof MR and Komdeur J 2018 Why and how the early-life environment affects development of coping behaviours. Behavioral Ecology and Sociobiology 72(3): 34. 10.1007/s00265-018-2452-3 29449757 PMC5805793

[r44] Litten JC, Drury PC, Corson AM, Lean IJ and Clarke L 2003 The Influence of Piglet Birth Weight on Physical and Behavioural Development in Early Life. Neonatology 84(4): 311–318. 10.1159/000073640 14593242

[r45] Lockwood R 2016 Ethology, ecology and epidemioloy of canine aggression. The Domestic Dog: Its Evolution, Behaviour and Interactions with People pp 160–181. Cambridge University Press: Cambridge, UK.

[r46] Lord K 2013 A Comparison of the Sensory Development of Wolves (*Canis lupus lupus*) and Dogs (*Canis lupus familiaris*). Ethology 119(2): 110–120. 10.1111/eth.12044

[r47] Luescher AU and Reisner IR 2008 Canine Aggression Toward Familiar People: A New Look at an Old Problem. Veterinary Clinics of North America: Small Animal Practice 38(5): 1107–1130. 10.1016/j.cvsm.2008.04.008 18672156

[r48] Malm K and Jensen P 1997 Weaning and Parent-Offspring Conflict in the Domestic Dog. Ethology 103: 653–664.

[r49] Mugnier A, Chastant S, Saegerman C, Gaillard V, Grellet A and Mila H 2021 Management of Low Birth Weight in Canine and Feline Species: Breeder Profiling. Animals 11(10): 2953. 10.3390/ani11102953 34679974 PMC8532740

[r50] Nishi M 2020 Effects of Early-Life Stress on the Brain and Behaviors: Implications of Early Maternal Separation in Rodents. International Journal of Molecular Sciences 21(19): 7212. 10.3390/ijms21197212 33003605 PMC7584021

[r51] Overall K 2013 Manual of Clinical Behavioral Medicine for Dogs and Cats. Elsevier: St Louis, MO, USA

[r52] Pal SK, Roy S and Ghosh B 2021 Pup rearing: The role of mothers and allomothers in free-ranging domestic dogs. Applied Animal Behaviour Science 234: 105181. 10.1016/j.applanim.2020.105181

[r53] Passalacqua C, Marshall-Pescini S, Barnard S, Lakatos G, Valsecchi P and Prato Previde E 2011 Human-directed gazing behaviour in puppies and adult dogs, *Canis lupus familiaris*. Animal Behaviour 82(5): 1043–1050. 10.1016/j.anbehav.2011.07.039

[r54] Pierantoni L, Albertini M and Pirrone F 2011 Prevalence of owner-reported behaviours in dogs separated from the litter at two different ages. Veterinary Record 169. 10.1136/vr.d4967 21865608

[r55] Qualtrics 2020 Qualtrics*. (Version 2023).* Qualtrics: Provo, Utah, USA. https://www.qualtrics.com (accessed 29th May 2026).

[r56] Range F, Ritter C and Virányi Z 2015 Testing the myth: tolerant dogs and aggressive wolves. Proceedings of the Royal Society B: Biological Sciences 282(1807): 20150220. 10.1098/rspb.2015.0220 PMC4424647 25904666

[r57] Rausch Q, Sriananthan O, Gibson E, Lo G, Widowski T, Coe J, Jacobs J and Niel L Factors influencing puppy competitive behaviour during the beginning and middle of a nursing bout in the domestic dog (*Canis lupus familiaris*). *Applied Animal Behaviour Science*, in review.

[r58] Reisner IR and Shofer FS 2008 Effects of gender and parental status on knowledge and attitudes of dog owners regarding dog aggression toward children. Journal of the American Veterinary Medical Association 233(9): 1412–1419. 10.2460/javma.233.9.1412 18980492

[r59] Rödel HG, Bautista A, García-Torres E, Martínez-Gómez M and Hudson R 2008 Why do heavy littermates grow better than lighter ones? A study in wild and domestic European rabbits. Physiology & Behavior 95(3): 441–448. 10.1016/j.physbeh.2008.07.011 18675835

[r60] Santos NR, Beck A, Maenhoudt C and Fontbonne A 2020 Influence of ADAPTIL® during the Weaning Period: A Double-Blinded Randomised Clinical Trial. Animals 10(12): 2295. 10.3390/ani10122295 33291607 PMC7761923

[r61] Sarkar R, Bhowmick A, Dasgupta D, Banerjee R, Chakraborty P, Nayek A, Sreelekshmi R, Roy A, Sonowal R, Mondal AB and Bhadra A 2023 Eating smart: Free-ranging dogs follow an optimal foraging strategy while scavenging in groups. Frontiers in Ecology and Evolution 11: 1099543. 10.3389/fevo.2023.1099543

[r62] Scott JP and Fuller J 1965 Genetics and the Social Behaviour of the Dog. University of Chicago Press: Chicago, USA.

[r63] StataCorp 2021 *Stata Statstical Software: Release 17. (Version 17).* StataCorp LLC: College Station, TX, USA.

[r64] Storozuk A, Ashley M, Delage V and Maloney EA 2020 Got Bots? Practical Recommendations to Protect Online Survey Data from Bot Attacks. The Quantitative Methods for Psychology 16(5): 472–481. 10.20982/tqmp.16.5.p472

[r65] Udell MAR, Ewald M, Dorey NR and Wynne CDL 2014 Exploring breed differences in dogs (Canis familiaris): does exaggeration or inhibition of predatory response predict performance on human-guided tasks? Animal Behaviour 89: 99–105. 10.1016/j.anbehav.2013.12.012

[r66] Vaterlaws-Whiteside H and Hartmann A 2017 Improving puppy behavior using a new standardized socialization program. Applied Animal Behaviour Science 197: 55–61. 10.1016/j.applanim.2017.08.003

[r67] Veenema AH 2009 Early life stress, the development of aggression and neuroendocrine and neurobiological correlates: What can we learn from animal models? Frontiers in Neuroendocrinology 30(4): 497–518. 10.1016/j.yfrne.2009.03.003 19341763

[r68] Vieira De Castro AC, Fuchs D, Morello GM, Pastur S, De Sousa L and Olsson IAS 2020 Does training method matter? Evidence for the negative impact of aversive-based methods on companion dog welfare. PLOS ONE 15(12): e0225023. 10.1371/journal.pone.0225023 33326450 PMC7743949

[r69] Welfare in Pet Trade 2020 *Responsible dog breeding guidelines.* EU Platform on Animal Welfare. https://food.ec.europa.eu/system/files/2020-11/aw_platform_plat-conc_guide_dog-breeding.pdf (accessed 19 June 2026).

[r70] Wright JC 1980 The development of social structure during the primary socialization period in German shepherds. Developmental Psychobiology 13(1): 17–24. 10.1002/dev.420130104 7353718

[r71] Zhu B, Wang F, Su X, Lu Y and Zhang H 2021 Effect of different amount of food and female resource on competitive strategy and agonistic behaviour of swimming crab (*Portunus trituberculatus*). Aquaculture 536. 10.1016/j.aquaculture.2021.736471

[r72] Ziv G 2017 The effects of using aversive training methods in dogs—A review. Journal of Veterinary Behavior 19: 50–60. 10.1016/j.jveb.2017.02.004

