# Supplementary material for: Resource management practices of Canadian and American domestic dog breeders and associations with puppy competitive behaviour: A cross-sectional study
Source: Anim Welf. 2026 Jul 22;35:e42. doi: 10.1017/awf.2026.10098 (PMC13419449; doi:10.1017/awf.2026.10098)
Supplement: Rausch et al. supplementary material [file S0962728626100980sup001.pdf]

Resource management practices of Canadian and American domestic dog breeders and associations with puppy competitive behaviour: A cross-sectional study: Supplementary material

Quinn Rauschhttps://orcid.org/0000-0001-8504-6611[1, 4], Samantha White[1], Tina Widowski[2], Jason Coe[1], Jacquelyn Jacobs[3], Lee Niel[1]

[1] Department of Population Medicine, Ontario Veterinary College, University of Guelph, Guelph, Canada

[2] Department of Animal Biosciences, Ontario Agricultural College, University of Guelph, Guelph, Canada

[3] Department of Animal Science, Michigan State University, East Lansing, US

[4] Messerli Research Institute, University of Veterinary Medicine Vienna, Veterinärplatz 1 (Building AZ), 1210 Vienna, Austria

Author for correspondence: Quinn Rausch, email: quinn.rausch@vetmeduni.ac.at

1. **Do you have a female dog that has had a litter of puppies in the last 3 years?** (Choose from: Yes; No)
2. **By legal definition, are you the owner of the female dog that had a litter of puppies?** (Choose from: Yes; No)
3. **What country do you currently live in?** (Choose from: Canada; United States; Other)
4. **Which province/territory do you currently live in?** (Choose one selection from dropdown list: all Canadian provinces)
5. **Which state do you currently live in?** (Choose one selection from dropdown list: all American states)
6. **Did you breed dogs before the beginning of the COVID-19 pandemic (April 1st, 2020)?** (Choose from: Yes, I bred before the COVID-19 pandemic; No, I started breeding during the COVID-19 pandemic)

Breeding Demographics

7. **How many different dog breeds have you bred at your facility in the past three years?** (Choose one selection from dropdown list: 1-100)
8. **Please select the breed that you have been most involved with breeding over the past three years. Please answer the rest of the survey about only this breed.** (Choose one selection from dropdown list: AKC recognized breeds)
9. **Which of the following best describes your involvement in dog breeding over the past three years?** (Choose from: My dog got pregnant and had a litter of puppies, but it was unplanned; I have one or more breeding dams that I purposely breed)
10. **Are you currently a member of a breed club that is associated with Canadian Kennel Club?** (Choose from: Yes; No; Unsure)
11. **Are you currently a member of a breed club that is associated with American Kennel Club?** (Choose from: Yes; No; Unsure)
12. **Are you currently a member of the United Kennel Club?** (Choose from: Yes; No; Unsure)
13. **Was your last litter of puppies registered with the American, United or Canadian Kennel Club?** (Choose from: Yes; No; Unsure)
14. **How many breeding dams do you own that are actively involved in your current breeding program?** (Choose one selection from dropdown list: 0-30+)
15. **On average, how many litters of puppies do you raise in total per year?** (Choose one selection from dropdown list: 0-250+)
16. **How many years have you been involved in dog breeding?** (Choose one selection from dropdown list: Less than 1-100 or greater)
17. **For the primary breed you selected above, what is the intended purpose of the dogs that you breed?** (Select all that apply: Companionship (e.g., pet); Working - assistance (e.g., service, guide, assistance); Working - on farm (e.g., on farm guarding and herding); Working - protection (e.g., military, detection, police); Competition – Conformation; Competition - Performance (e.g., racing, agility, obedience, scent work, hunting, field trials); Other)
18. **How many puppies are in your average litter?** (Choose one selection from dropdown list: Less than 1-20+)
19. **Where do you house the dam with puppies?** (Choose from: At my facility in the house I live in; At my facility inside a building I do not live in; At my facility outside; Not at my facility)

Feeding Practices

20. **During nursing, do all puppies in the litter typically nurse at the same time?** (Choose from: Yes; No; Unsure)
21. **Do you ever observe competition between puppies around access to particular nipples or to the dam in general during nursing (e.g., pushing with head or paws, displacement of puppies off nipples, body blocking, growling, nipping, biting)?** (Choose from: Yes; No)
22. **Do you ever intervene with the litter during nursing (e.g., touch, move, adjust puppies)?** (Choose from: Yes; No)
23. **If you do intervene with nursing, when do you intervene, how do you intervene, and why?** (Open text box)
24. **How are puppies weaned?** (Choose from: Naturally, up to the dam; Human intervention; Other (please specify))
25. **On average, when do you (or the dam) start the weaning process?** (Choose one selection from dropdown list: Never - >16 weeks)
26. **On average, when are puppies completely weaned?** (Choose one selection from dropdown list: Never - >16 weeks)
27. **When do you typically introduce solid food (anything except milk) to the puppies?** (Choose one selection from dropdown list: Never - >16 weeks)
28. **In your experience, when are puppies typically interested in solid food?** (Choose one selection from dropdown list: Never - >16 weeks)
29. **How is solid food typically presented to the puppies?** (Choose from: At least one feeder/bowl per puppy (puppies do not share); Multiple puppies per one feeder/bowl (puppies share); Other)
30. **When feeding solid foods, do all puppies in the litter typically eat at the same time?** (Choose from: Yes; No; Unsure)
31. **After solid food is introduced, is it always available to puppies?** (Choose from: Yes, always available; No, only at limited times; No solid food offered
32. **How many times per day is solid food provided?** (Choose one selection from dropdown list: 1 time–10+ times)
33. **What type of solid food is provided to the puppies when it is first introduced?** (Choose from: Wet food; Wet food mixed with water; Dry kibble; Dry kibble mixed with wet food; Dry kibble mixed with formula; Dry kibble mixed with water; Other (please specify)
34. **Do you do any of the following when puppies are actively feeding on solid food?** (Select all that apply: Remove food while puppy is feeding; Pet or handle puppy during feeding; Touch food and feeder while puppy is feeding; Add food or treats to feeder while puppy is feeding)
35. **Do you interact or intervene with puppies in any other ways while they are actively feeding on solid foods?** (Choose from: Yes (please elaborate); No)
36. **Do puppies ever perform any of the following behaviours towards HUMANS who approach while they are eating solid food?**
    a. Body blocking humans from food (Choose from: Yes; No; Unsure)
    b. Eating quicker than normal when humans are close (Choose from: Yes; No; Unsure)
    c. Grabbing food and moving away from human contact (Choose from: Yes; No; Unsure)
    d. Growling (Choose from: Yes; No; Unsure)
    e. Lunging, nipping, or biting (Choose from: Yes; No; Unsure)
    f. Pushing human contact away with nose or paw (Choose from: Yes; No; Unsure)
37. **If you see behaviours directed towards HUMANS around the feeder that you would like to discourage, how do you typically respond?** (Select all that apply: Continue interacting without changing approach; Stop interacting with the puppy; Deter puppy with physical or verbal correction (e.g., spray with water, startle with noise, use loud tone of voice, hold mouth or scruff etc.); Attempt to calm puppy with petting or speaking; Interrupt puppy and redirect attention

elsewhere (e.g. treat, play, toy etc.); Remove food from puppy's reach; My puppies do not typically show these behaviours; Other (please specify))

38. **Do puppies ever perform any of the following behaviours towards other puppies who approach while they are eating solid food?**
    a. Body blocking other puppies from food (Choose from: Yes; No; Unsure)
    b. Eating quicker than normal when other puppies are close (Choose from: Yes; No; Unsure)
    c. Grabbing food and moving away from other puppies (Choose from: Yes; No; Unsure)
    d. Growling (Choose from: Yes; No; Unsure)
    e. Lunging, nipping, or biting (Choose from: Yes; No; Unsure)
    f. Pushing other puppies away with nose or paw (Choose from: Yes; No; Unsure)
39. **If you see behaviours directed towards other PUPPIES around the feeder that you would like to discourage, how do you typically respond?** (Select all that apply: Provide additional feeders to deter competition; Deter puppy with physical or verbal correction (e.g., spray with water, startle with noise, use loud tone of voice, hold mouth or scruff etc.); Attempt to calm puppy with petting or speaking; Interrupt puppy and redirect attention elsewhere (e.g., treat, play, toy etc.); Remove food from puppy's reach; Separate puppies from each other; My puppies do not typically react negatively; Other (please specify))

Play Management

40. **When do you start introducing play objects (e.g., balls, stuffed animals, puzzles) to the puppies?** (Choose one selection from dropdown list: Never - >16 weeks)
41. **Which of the following play objects do you offer to young puppies (<12 weeks old) prior to sale/adoption? (**Select all that apply: Rope toys; Bone toys for chewing; Balls; Stuffed plush toys; Toys that squeak or make other noises; Puzzle or feeder toys (e.g., stuffed Kong, snuffle mat); Other (please specify))
42. **Do individual puppies seem to have preferences for particular toys?** (Choose from: Yes; No; Unsure)
43. **How many play objects are available per puppy?** (Choose from: One or more play objects per puppy; Multiple puppies per one play object; I do not provide play objects)
44. **Do puppies in the litter typically play at the same time?** (Choose from: Yes; No)
45. **Are play objects always available to puppies?** (Choose from: Yes, always available; No, only at limited times; I do not provide play objects)
46. **Do you do any of the following when puppies are playing with toys?** (Select all that apply: Remove toy while puppy is playing; Pet or handle puppy while playing; Touch toy while puppy is playing; Trade the puppy for another better toy or treat (trade-up))
47. **Do you interact with puppies in any other ways while they are playing with toys?** (Choose from: Yes; No)
48. **Do puppies ever perform any of the following behaviours towards HUMANS who approach while they are playing with toys?**
    a. Body blocking humans from toy (Choose from: Yes; No; Unsure)
    b. Grabbing toy and moving away from human contact (Choose from: Yes; No; Unsure)
    c. Growling (Choose from: Yes; No; Unsure)
    d. Lunging, nipping, or biting (Choose from: Yes; No; Unsure)
    e. Pushing human contact away with nose or paw (Choose from: Yes; No; Unsure)
49. **If you see behaviours directed towards HUMANS around toys that you would like to discourage, how do you typically respond?** (Select all that apply: Continue interfering without changing approach (if purposefully interfering for training purposes); Stop interfering (if purposefully interfering for training purposes); Deter puppy with physical or verbal correction (e.g. spray with water, startle with noise, use loud tone of voice, hold mouth or scruff etc.); Comfort with petting or speaking; Interrupt puppy and redirect attention elsewhere (e.g. treat,

play, toy etc.); Remove toy from puppy's reach; My puppies do not perform behaviours that I want to discourage)

50. **Do puppies ever perform any of the following behaviours towards other PUPPIES who approach while they are playing with toys?**
    a. Body blocking other puppies from toy (Choose from: Yes; No; Unsure)
    b. Grabbing toy and moving away from other puppies (Choose from: Yes; No; Unsure)
    c. Growling (Choose from: Yes; No; Unsure)
    d. Lunging, nipping, or biting (Choose from: Yes; No; Unsure)
    e. Pushing other puppies away with nose or paw (Choose from: Yes; No; Unsure)
    f. 'Tug of war' with other puppies over toys (Choose from: Yes; No; Unsure)
51. **If you see behaviours directed towards other PUPPIES around toys that you would like to discourage, how do you typically respond?** (Select all that apply: Provide additional toys to deter competition; Deter puppy with physical or verbal correction (e.g., spray with water, startle with noise, use loud tone of voice, hold mouth of scruff etc.); Comfort with petting or speaking; Interrupt puppy and redirect attention elsewhere (e.g., treat, food etc.); Remove toy from puppies' reach; My puppies do not perform behaviours that I want to discourage; Separate puppies from each other)

<u>COVID-19 Impacts</u>

52. **Since the onset of the COVID-19 pandemic, have your puppies' introduction and access to resources been impacted (e.g., limited access to supplies or specific food/toys)?** (Choose from: Yes; No; Unsure)
53. **Please explain briefly how the COVID-19 pandemic has impacted your puppies' introduction and access to resources.** (open textbox)

<u>General Demographics</u>

54. **What is your gender?** Choose from: Man; Woman; Non-binary; My gender identify is not listed above; Prefer not to answer)
55. **What is the highest level of education you have completed?** (Choose from: Elementary School; Secondary School; College Certificate or Diploma; Bachelor's Degree; Master's Degree; Doctor of Philosophy Degree; Professional Degree; Other (please specify); Prefer not to answer)
56. **What is your age (in years)?** (Choose one selection from dropdown list: 18-100)
57. **I live in a:** (Choose from: Rural property outside of a concentrated housing area; Village (<1000 people); Small town (1000 to 20,000 people); Large town (20,000 to 100,000 people); Small city (100,000 to 300,000 people); Large city (300,000 to 1 million people); Metropolis (>1 million people)

Table S1: Odds ratios, confidence intervals and p-values of fixed effects included in final logistic regression models based on data collected from Canadian and American dog breeder participants (n=293) in a puppy management survey between 2021 and 2022.

**Fixed Effects**	**Odds Ratio**	**95% CI**	***P*-value**
Model 1: food competition towards humans			
Nursing intervention (referent: no intervention)	0.27	0.13-0.55	<0.001
Add food into food bowl while puppy is eating (referent: no addition)	1.83	1.10-3.30	0.047
United States (referent: Canada)	2.85	1.50-5.30	0.001
Model 2: food competition towards littermates			
Herding breeds (referent: toy breeds)	5.68	1.56-20.69	0.008
Sporting breeds (referent: toy breeds)	3.13	0.88-11.20	0.079
Non-sporting breeds (referent: toy breeds)	1.80	0.40-8.07	0.443
Terrier breeds (referent: toy breeds)	1.83	0.46-7.32	0.394
Hound breeds (referent: toy breeds)	4.25	1.02-17.73	0.047
Working breeds (referent: toy breeds)	4.64	1.24-17.38	0.023
'Other' (mixed/cross) breeds (referent: toy breeds)	8.10	1.74-37.75	0.008
Model 3: toy competition towards humans			
Competitive behaviour towards humans around food (referent: no competitive behaviour)	3.43	1.86-6.36	<0.001
Preference for specific toys (referent: no preference)	2.93	1.64-5.23	<0.001
Remove toy while puppy is playing (referent: no removal)	2.28	1.37-3.82	0.002
Model 4: threatening or aggressive toy competition towards littermates			
Food competition towards humans (referent: no competition)	3.88	2.33-6.47	<0.001
Litter typically plays together (referent: don't play together)	0.51	0.29-0.91	0.023
Model 5: use of aversive-based training			
Competitive behaviour during suckling (referent: no competition)	2.34	1.17-4.68	0.017
Food competition towards humans (referent: no competition)	3.40	1.83-6.33	<0.001
Toy competition towards humans (referent: no competition)	2.27	1.29-4.00	0.004
